# Novel Chitosan-Silica Hybrid Hydrogels for Cell Encapsulation and Drug Delivery

**DOI:** 10.3390/ijms222212267

**Published:** 2021-11-12

**Authors:** Soher N. Jayash, Paul R. Cooper, Richard M. Shelton, Sarah A. Kuehne, Gowsihan Poologasundarampillai

**Affiliations:** 1 School of Dentistry, University of Birmingham, 5 Mill Pool Way, Edgbaston, Birmingham B5 7EG, UK; R.M.Shelton@bham.ac.uk (R.M.S.); S.A.Kuehne@bham.ac.uk (S.A.K.); 2 Department of Oral Sciences, Sir John Walsh Research Institute, Faculty of Dentistry, University of Otago, P.O. Box 56, Dunedin 9054, New Zealand; P.Cooper@otago.ac.nz

**Keywords:** chitosan, thiolated chitosan, organic-inorganic hybrid hydrogel, sol-gel process, cell encapsulation, drug delivery

## Abstract

Hydrogels constructed from naturally derived polymers provide an aqueous environment that encourages cell growth, however, mechanical properties are poor and degradation can be difficult to predict. Whilst, synthetic hydrogels exhibit some improved mechanical properties, these materials lack biochemical cues for cells growing and have limited biodegradation. To produce hydrogels that support 3D cell cultures to form tissue mimics, materials must exhibit appropriate biological and mechanical properties. In this study, novel organic-inorganic hybrid hydrogels based on chitosan and silica were prepared using the sol-gel technique. The chemical, physical and biological properties of the hydrogels were assessed. Statistical analysis was performed using One-Way ANOVAs and independent-sample *t*-tests. Fourier transform infrared spectroscopy showed characteristic absorption bands including amide II, Si-O and Si-O-Si confirming formation of hybrid networks. Oscillatory rheometry was used to characterise the sol to gel transition and viscoelastic behaviour of hydrogels. Furthermore, in vitro degradation revealed both chitosan and silica were released over 21 days. The hydrogels exhibited high loading efficiency as total protein loading was released in a week. There were significant differences between TC_2_G and C_2_G at all-time points (*p* < 0.05). The viability of osteoblasts seeded on, and encapsulated within, the hydrogels was >70% over 168 h culture and antimicrobial activity was demonstrated against *Pseudomonas aeruginosa* and *Enterococcus faecalis*. The hydrogels developed here offer alternatives for biopolymer hydrogels for biomedical use, including for application in drug/cell delivery and for bone tissue engineering.

## 1. Introduction

Skeletal tissue defects resulting from disease, congenital deformities or trauma are often treated by surgery using autografts, allografts and/or xenografts. Autografts harvested from the patient remain the gold standard but are limited by availability and donor site morbidity [1]. While allografts and xenografts are readily available in sufficient quantity these materials may cause risk of infection transmission, unpredictable bone formation and immunogenic responses [2,3,4]. Tissue engineering and regenerative medicine approaches have been proposed to develop implantable graft constructs that are as potent as autografts but overcome current limitations [5]. Natural polymer hydrogels that imitate the extracellular matrix (ECM) of tissues are promising materials for the therapeutic delivery of drugs, proteins and cells for tissue repair and regeneration [6,7]. Injectability of such hydrogel systems also allows for minimally invasive surgery to fill defects [8,9].

Different types of hydrogels are being developed and constructed from natural or synthetic materials. Hydrogels made from naturally derived biopolymers, such as collagen, hyaluronic acid and gelatin, were the first to be identified as having the potential to mimic the ECM [10]. Also, chitosan based hydrogels were shown to enhance new bone formation [11]. These naturally derived hyrogels have physiochemical advantages including the provision of an aqueous environment that is not cytotoxic and enables cell proliferation. However, these gels exhibit poor mechanical strength (e.g., both compressive and tensile) and batch-to-batch variation which makes production and optimisation challenging [12,13,14]. Conversely, synthetic hydrogels based on polyethylene glycol (PEG) have enhanced mechanical strength compared with naturally derived hydrogels which provides stability to support cells during 3D culture [15]. However, relatively slow degradation may be a limitation [16] as well as the lack of ligand chemistry to enable cell instructive signals [17,18]. Peptide-based hydrogels overcome many of these problems and consequently have attracted significant interest in the biomedical field, although suffer from various drawbacks, including solubility issues, burst release, low bioavailability due to high clearance or metabolism, enhanced degradation and nonspecific distribution [19,20]. There is a serious need for new hydrogel biomaterials with suitable mechanical and biochemical properties to support cells in a 3D environment and address these issues.

Hybrid materials with molecularly interpenetrating networks of organic and inorganic phases can be classified based on the type of bonding present between the different moieties [21]. Covalently crosslinked hybrids (Class II), the degree of crosslinking and the proportion of organic to inorganic components, provide excellent control of mechanical properties and degradation behaviour [22,23,24]. Hybrid materials for biomedical applications often employ a natural polymer which presents ligands that provide biochemical cues for cells [25], whilst, silica derived from organosilanes forms the inorganic component. Due to the diversity of natural polymers and the versatility of organosilane chemistry, many types of hybrid materials have been developed for biomedical applications [26,27,28,29]. Incorporation of silica in hybrid hydrogels leads to the formation of a physical network and increases the strength of adsorption of polymer network that will be important for the mechanical properties, as both physical adsorption and chemical covalent bonds are present. Such chemically cross-linked gels containing polymers on silica nanoparticle materials provide a generic route for improving hydrogel properties [30,31]. Chitosan-silica hybrids show much promise due to being biodegradable, non-antigenic, nontoxic and promoting certain biological processes [32,33]. Furthermore, chitosan can be functionalised with 2-iminothiolane hydrochloride to produce thiolated chitosan (TC) with thiol functional side groups [34] which serve to improve permeation and mucoadhesive properties of chitosan. Hydrogen bonding and hydrophobic interactions also play important roles in the mucoadhesion of chitosan. The enhancement in mucoadhesion is based on the formation of disulfide bridges with mucus glycoproteins and the formation of covalent bonds between the polymer and the mucus layer [34,35,36,37,38].

Organic-inorganic hybrids have previously been synthesised using alkoxysilanes as the inorganic precursors, most notably tetraethoxysilane (TEOS) [39,40,41]. These undergo sol to gel transition releasing ethanol when reacted in acidic or alkaline aqueous solutions. Ethanol is a denaturant, thus hybrid hydrogels intended for delivery of therapeutics and/or cell encapsulation cannot be synthesised using alkoxysilanes. Non-cytotoxic inorganic precursors that undergo sol to gel transition within physiologically benign conditions are therefore desirable [42,43]. Synthetic 3D materials are required as grafts for clinical applications to regenerate healthy tissues by combating infection, promoting vascularisation, accelerating wound healing and bone formation. This work produced a novel synthetic graft platform for use in tissue engineering by developing advanced organic-inorganic hybrid biomaterials. Organic-inorganic hybrid hydrogels were developed using the ‘soft-chemistry’ sol-gel process to take advantage of the physiochemical properties of the biopolymers. The predictable and tuneable nature of synthetic silica materials allows ‘dial-in’ degradation, bioactivity and mechanical properties via the manipulation of relative chitosan/bioactive-silica compositions and the strength of interactions. Also, this study presents the development and characterisation of novel chitosan-silica hybrid hydrogels employing polyol-modified silanes, providing a new class of biologically suitable silica precursor for bone tissue engineering.

## 2. Results

Organic-inorganic hybrid hydrogels with covalent crosslinking employing GPTMS were produced. These were labelled as C-chitosan, TC-thiolated chitosan and C_1_G, C_2_G and C_10_G or TC_1_G, TC_2_G and TC_10_G representing chitosan or thiolated chitosan crosslinked with 1 mole of GPTMS for X moles of glucosamine monomer. Increasing X gives decreasing crosslinking.

### 2.1. Functionalisation of Chitosan and Characterisation of the Hybrid Structure

**Figure 1:**
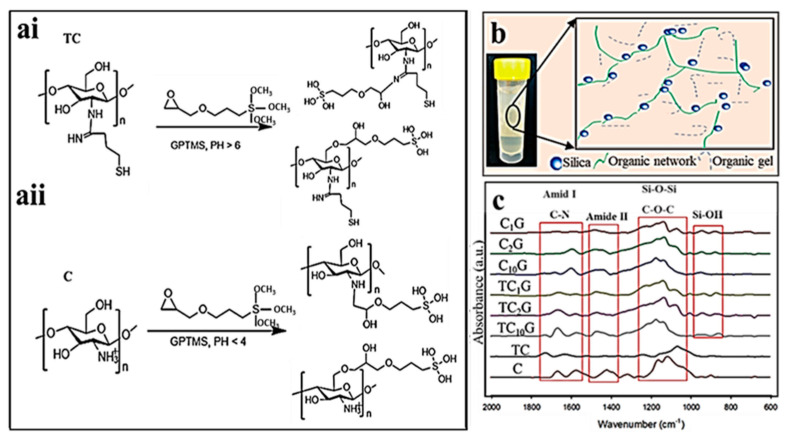
(**a**) Schematic showing the reaction sequences and the chemical structures of thiolated chitosan (**ai**) and chitosan (**aii**) needed for hydrogel preparation. (**b**) A schematic image of the distribution of globular particles of the branched 3D network of silica and chitosan on the hybrid hydrogel. (**c**) FTIR spectra of hybrid hydrogels with varying organic/inorganic weight ratios showing amide I and II, Si-O-Si and Si-ONB bands.

Functionalisation of chitosan with GPTMS and the possible structures that could form are illustrated in Figure 1a,b. The hydrogels fabricated with the various components were investigated using FTIR and ^1^H NMR. Bands corresponding with amide I (C=O: vs. 1600–660 cm^−1^), amide II (N-H: v_b_ 1470–1590 cm^−1^), Si-O-Si (v_b_ 1060–1180) and Si-O^NBO^ (v_b_ 940–970) (Figure 1c) were used to ascertain the reaction of thiolated chitosan or chitosan with GPTMS [44,45]. FTIR spectra revealed differences between chitosan and thiolated chitosan as Amide I and Amide II bands appeared shifted to higher wavenumbers once thiolated. Furthermore, with an increasing amount of coupling agent in the functionalised polymer (C_x_G and TC_x_G) an higher intensity of Si-O-Si and Si-O^NB^ vibrations were observed suggesting an enhanced degree of inorganic silica condensation and crosslinking.

**Figure 2:**
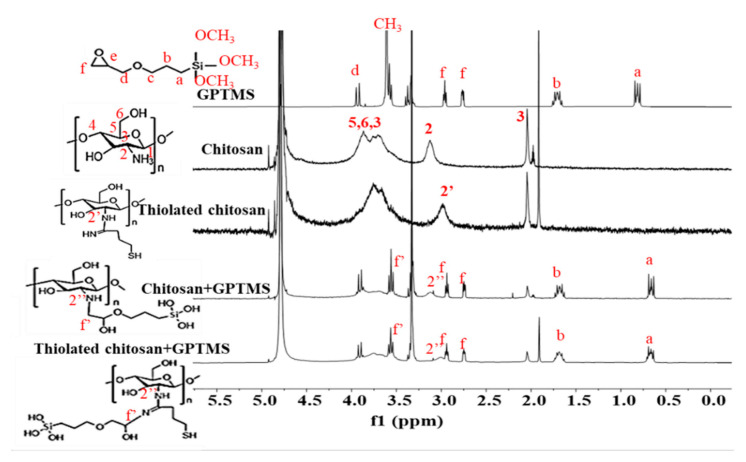
H-NMR spectra of GPTMS and chitosan or thiolated chitosan before and after functionalisation with GPTMS for 24 h in ratio 1:4.

^1^H NMR spectra (Figure 2) for chitosan revealed peaks between 3.0 and 5.0 ppm, which were ascribed to the protons of the glucosamine unit [46,47]. The peak at 2.1 ppm was due to the protons of the methyl group in the N-acetylglucosamine unit. The sulfhydryl hydrogen was relatively easily replaced by deuterium present within solvent D_2_O in NMR spectra, consequently, the thiol peak was difficult to distinguish in the thiolated chitosan spectrum [48]. However, the new chemical shift corresponding with the proton on the carbon of amine appeared in thiolated chitosan (δ^1^H 2.9 ppm). Peaks in GPTMS-functionalised polymers (chitosan and thiolated chitosan) and GPTMS spectra attributed to protons of the epoxide ring were identified between δ^1^H 2.9 and δ^1^H 2.7 ppm which suggested a large number of epoxides remained intact. In addition, a new peak was observed in the 3.5 ppm region, which was attributed to the reaction of the epoxide ring with the primary amine of chitosan to form a secondary amine as previously observed by Connell et al. [49]. ^1^H NMR also showed the silanes had completely hydrolysed following the functionalisation reaction due to the loss of methyl groups on the GPTMS.

### 2.2. In Vitro Degradation of the Hydrogel

In vitro biodegradation of hydrogels was studied by monitoring weight loss and analysing substances eluted into PBS with and without lysozyme over time. The degradation rate was the slowest in C_1_G (<5%) after 1 h and in C_2_G after 24 h (<30%) (Supplementary Figure S1). The degradation rates of C_10_G and TC_10_G were more than 70% at 24 h and up to 90% by weight of hydrogel was degraded by 21 days. Hydrogel weight-loss after 504 h showed that C_2_G and TC_2_G had the slowest rate of degradation; consequently, hydrogels with this ratio were selected for further detailed analysis.

**Figure 3:**
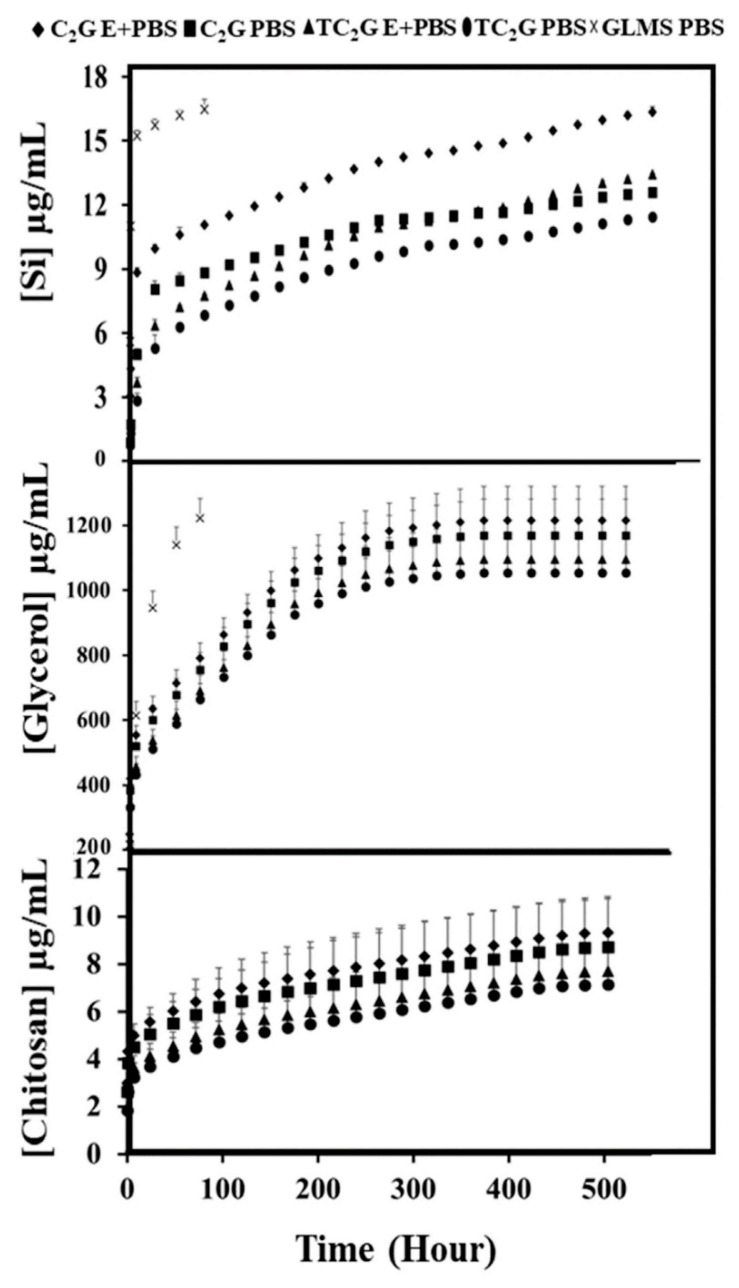
Accumulation of silica, glycerol and chitosan degradation products released in degradation solution of TC_2_G and C_2_G up 504 h. E+PBS: degradation solution containing lysozyme, PBS: only PBS.

Degradation products from the hydrogels were further analyzed. Burst release was observed in the first few days followed by controlled release (Figure 3). Soluble silica concentration as a function of immersion time in PBS was measured using ICP-AES and the degraded organics, glycerol and chitosan (released in the form of glucosamine (GA) and N-acetyl-D-glucosamine (N-Ac-GA)), were quantified using HPLC. The GLMS hydrogel (used as control) was completely degraded or became unstable after 3 days. The quantity of degradation products released from hydrogels in PBS containing lysozyme was greater than in PBS alone. Two modes of silica release from hydrogels were observed, initial burst release, followed by a controlled release. Burst release of silica occurred in the C_2_G hydrogel at 7 h while the TC_2_G hydrogel demonstrated a slow release. TC_2_G immersed in PBS demonstrated the slowest rate of Si release, followed by TC_2_G immersed in PBS containing lysozyme where Si release remained at a relatively low level (0.3 ± 0.2 µg/mL per day) from 72 to 504 h. The maximum total concentration of silica released at 21 days was 16 µg/mL from C_2_G immersed in PBS with lysozme (supporting information Table S1).

HPLC analysis showed the retention time of glycerol was 2.78 min while GA and N-Ac-GA derivatives were detected in two peaks between 5 and 8 min compared with standards. The cumulative degradation products of glycerol shown in Figure 3 revealed 3 modes of release, initial burst release, followed by rapid release and then a plateau region. The highest release of glycerol occurred from the GLMS hydrogel within 96 h. Glycerol showed burst release up until 24 h followed by a controlled release at 189 h and a plateau profile until 504 h. The glycerol released from the TC_2_G and C_2_G hydrogels in the first week occurred rapidly and was responsible for the majority of the weight loss from the hydrogels. The highest accumulated concentration release of glycerol was 1214 µg/mL at 21 days (supporting information Table S2). Chitosan release followed a similar trend to that of silica release (Figure 3) as TC_2_G hydrogels exhibited the slowest release. Chitosan showed burst release until 24 h followed by controlled release up to 504 h. The highest accumulation concentration released was 10 µg/mL at 21 days. There was a significant difference between chitosan monomers released in enzymatic solution compared with PBS only (*p* < 0.05) in the first hour, after that time-point no significant difference was observed (*p* > 0.05) (supporting information Table S3).

### 2.3. Rheological Properties of Hybrid Hydrogels

**Figure 4:**
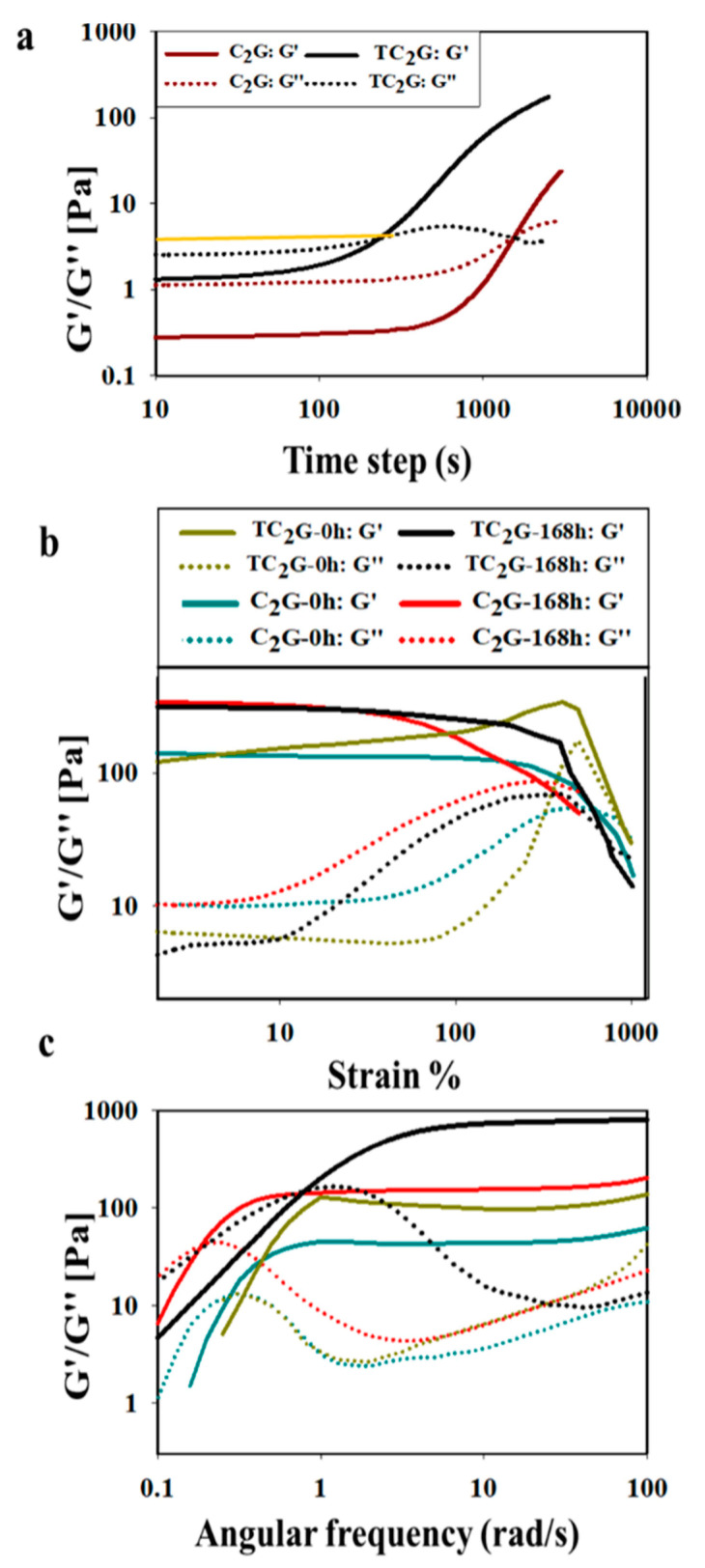
(**a**) Time dependence of the storage (G′) and loss (G″) moduli for C_2_G and TC_2_G. The gel point of TC2G was around 243s and 1627s for C_2_G and both gels had an identical modulus at the crossover-point. (**b**) Loss modulus (G′), storage modulus (G″) as a function of strain for TC_2_G and C_2_G immediately after gelling and one week after. (**c**) Loss modulus (G′), storage modulus (G″) as a function of frequency for TC_2_G and C_2_G immediately after gelling (G′/G′′/complex viscosity 1) and one week after gelling. TC_2_G has the highest storage moduli over one week.

Viscoelastic properties of the hybrids in liquid and gelled states were measured using a parallel plate oscillation rheometer. The gelling time for hybrids was performed at a constant frequency (10 rad/s) and strain (1%) sweeps were performed to assess sol to gel transition. Chitosan and thiolated-chitosan formulations exhibited low viscosity with thiolated chitosan exhibiting higher viscosity than chitosan. This coincided with a higher loss modulus (G″) than storage modulus (G′) in the liquid state. Both G′ and G″ increased rapidly as gelation proceeded; the build-up rate of G′was much higher than that of G″. The different rates led to a crossover of G′ and G″, which could be defined as the gel point (G′ = G″), which was approximately 243 ± 18 s for the TC_2_G hydrogel and 1627 ± 98 s for the C_2_G hydrogel (Figure 4a). Interestingly, both gels had an identical storage modulus at the crossover-point.

Strain sweeps at a constant frequency (10 rad/s) were performed on the hybrid hydrogels immediately or one week after gelation to determine linear-viscoelastic (LVE) regions (Figure 4b). The storage modulus of the TC_2_G hydrogel doubled from 160 to 320 Pa at 10% strain immediately and one week after gelling, respectively. Whilst for the C_2_G hydrogel, G′increased from 130 to 334 Pa at 10% strain immediately and one week after gelling, respectively. At the crossover point (flow point), the storage modulus of TC_2_G was 55 Pa and 40 Pa, immediately after gelling and one week after gelling, respectively. Whereas the storage modulus of C_2_G was 32 Pa and 89 Pa after gelling and one week after, respectively (Figure 4b). The flow point is an effective parameter that can be used to show the ability of the hydrogel to maintain its structural stability and prevent particle aggregation [50]. The highest flow point value was obtained for TC_2_G after gelling, which showed the least propensity to flow. Change in G′, G″ and complex viscosity of TC_2_G and C_2_G as a function of frequency at a constant strain of 1% is shown in Figure 4c. The storage modulus for all of the hydrogels exhibited a plateau in the range 1–100 rad/s, which was indicative of a stable cross-linked network. This G′-frequency independent feature was also indicative of a solid-like behaviour and pointed to the strength of the hydrogel, which was highest for TC_2_G amongst all the compositions tested. In the frequency test, G′ for TC_2_G and C_2_G immediately after gelling were 141 Pa and 60 Pa and were 790 and 202 Pa one week after gelling, respectively. The loss tangent (tanδ), which is the ratio of G″ to G′, indicated the overall viscoelasticity of the material and was a measure of the ratio of viscous to elastic conversation of energy during deformation. Tan δ for C_2_G and TC_2_G were <0.1 and <0.02, respectively, suggesting both hydrogels were elastic. These observations confirmed that the chitosan-silica hybrid hydrogels were stable and highly crosslinked.

### 2.4. Protein Release from Hybrid Hydrogels

**Figure 5:**
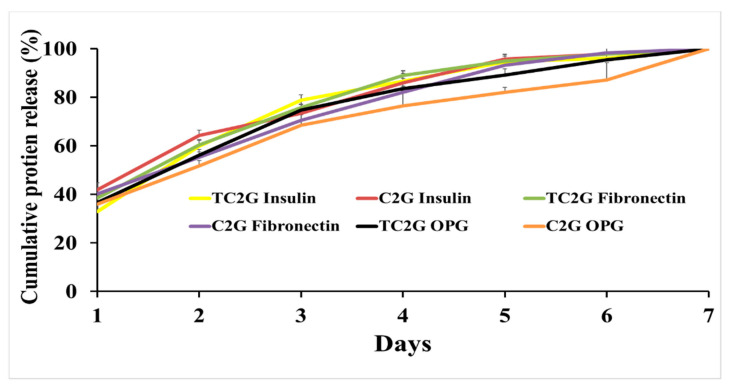
In vitro release of human insulin, fibronectin and OPG from C_2_G and TC_2_G over a 7-day period. The total protein loading in hydrogels was released in the first week. Triplicates for each hydrogel were analysed and means are shown.

The in vitro release kinetics profile of human insulin, fibronectin and OPG from TC_2_G and C_2_G hydrogels were performed in PBS at 37 °C and data are shown in Figure 5. Both hydrogels demonstrated that more than 30% of total proteins were released after the first day, the release was continuous with more than 30% being released up to 7 days after removing the protein released during the previous day. The total protein loading in 10 mg of hydrogels was 40 ng/mL, and on the first day, the amount of protein released from the hydrogel was <13 ng/mL. The total protein amount was released within the first week. There was no significant difference between the different proteins or hydrogels analysed.

### 2.5. Assessment of Cell Viability Seeded on/within Hybrid

**Figure 6:**
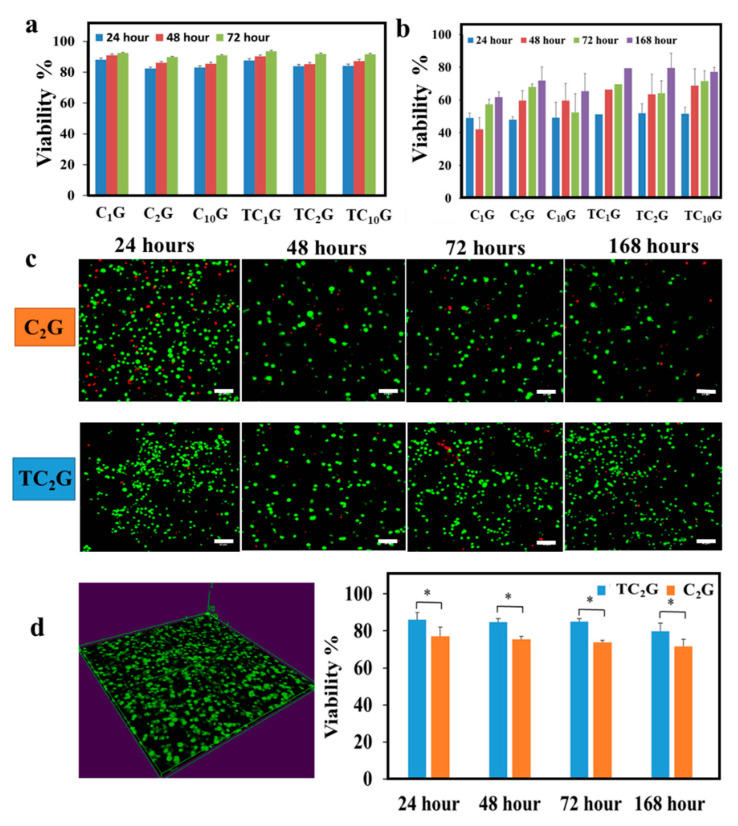
Effect of the different hydrogels on viability of osteoblasts seeded on these surfaces using the direct contact method up to 168 h (**a**) and the indirect contact method up to 72 h (**b**). (**c**): Representative confocal laser microscopic images stained using the live/dead assay for osteoblasts encapsulated in C_2_G and TC_2_G for 24, 48, 72 and 168 h (Scale bar is 50 µm). The green colour indicated viable cells and the red colour indicated non-viable cells. (**d**) image of hybrid hydrogels showing cells were encapsulated and evenly distributed within the hybrid hydrogels. Viability percentages of encapsulated osteoblasts in hydrogels at 24, 48, 72 and 168 h. The viability of cells was ≥70% at 168 h. Data are expressed as mean ± SD (n = 3). * denotes significant difference in viability percentage (*p* < 0.05) identified between the two hydrogels.

The viability of osteoblasts (SaOs-2 cells) treated with media extracts compared with cells seeded on wells with basal media was greater than 80% over the 72 h culture period (Figure 6a). When cells were directly seeded on the hydrogels, all hydrogels exhibited limited cytotoxicity as osteoblast viability remained at <70% until 72 h compared with controls (Figure 6b). Osteoblast viability increased gradually with increasing culture time. The viability of cells on thiolated chitosan-silica hybrids reached ~80% by 168 h. A LIVE/DEAD assay was also used to visualise the distribution of living and dead cells in the hydrogels at different time points for the 3D culture system. Relatively few dead cells were apparent and the number of live cells was much higher at 7 days (Figure 6c). The viability of cells encapsulated within hybrid hydrogels was ≥80% in TC_2_G and ≥70% in C_2_G for culture times up to 168 h (Figure 6d). There was a significant difference between TC_2_G and C_2_G materials at all-time points (*p* < 0.05). Statistical analysis of data obtained for the different hydrogels on the viability of osteoblasts are summarized in supporting information Table S4.

**Figure 7:**
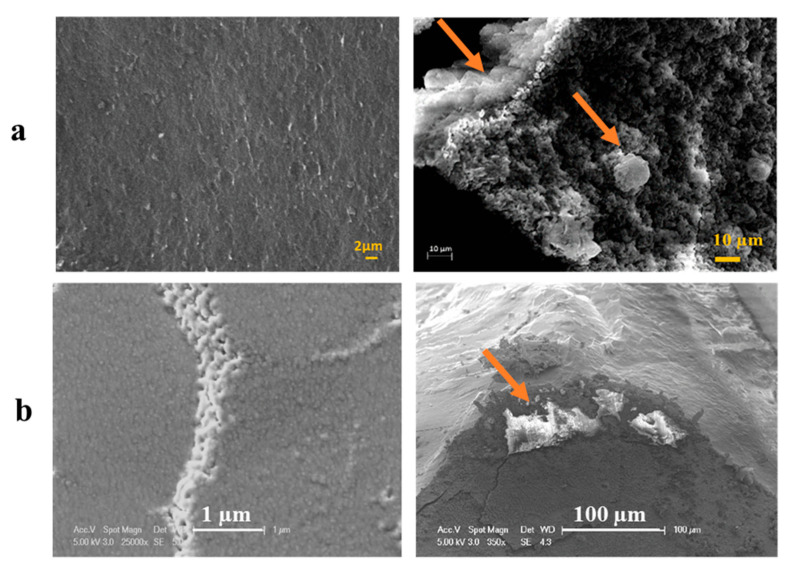
(**a**) SEM micrographs of C_2_G hydrogels without cells and with cells at 168 h after encapsulation. (**b**) Cryo-SEM image of TC_2_G showing the non-porous structure of the hydrogel and osteoblasts inside TC_2_G at 168 h (Scale bar = 100 μm). The arrows indicate cells in the hydrogel.

The morphology of the cells seeded in the hydrogels was assessed using SEM (Figure 7a). SEM images revealed the interaction of cells with the surrounding hydrogel matrix and protruding cell clusters from the hydrogels. Cells appeared attached to the hydrogel surface or encapsulated within the hydrogel. Also, SEM imaging revealed that the hydrogels were amorphous solids lacking pores or ordered structure. Cryo-SEM images show that the morphology of the organic-inorganic hybrid hydrogel after 168 h of cell culture was represented by a matrix of evenly distributed agglomerated particles. The gels were smooth and non-porous and the globular structures on the sample surface exhibited the formation of a 3D network of Si–O–Si bonds. Cryo-SEM micrographs of the fracture surface of TC_2_G hydrogels showed cells encapsulated in the hydrogel matrix (Figure 7b).

### 2.6. Assessment of Antibacterial Activities of Hybrid Hydrogels

**Figure 8:**
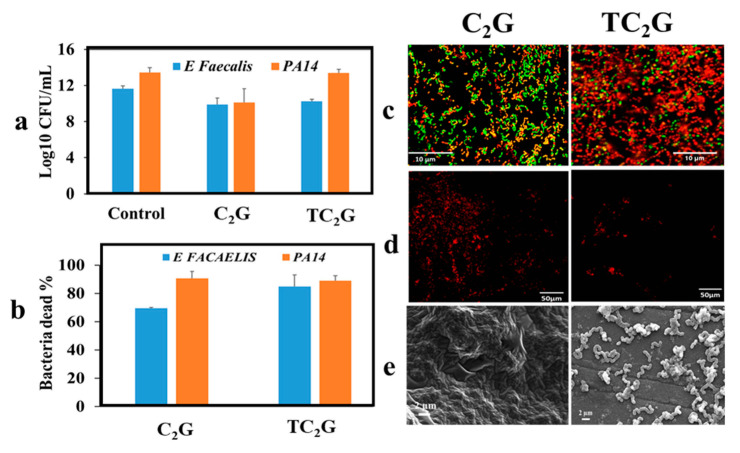
(**a**) Antimicrobial activity of hydrogels. Log_10_ reduction of bacteria (mean log CFU/mL of x experiments) after 24 h incubation on hydrogel and CFU plating. The control represented only bacteria without incubation on hydrogel. (**b**) quantitative data of the effect of hybrid hydrogels on the viability of bacteria stained with LIVE/DEAD^®^ Bacterial Viability Kit showing more >70% of bacteria dead after incubation with the hydrogels for 24 h. (**c**) Representative confocal microscopy images of bacteria on the surface of hydrogels after 24 h incubation period. Fluorescence images showing live (green) and dead (red) bacteria. (**d**) Fluorescence images (Live/dead stain) showing bacteria are dead in the hydrogels after 24 h. (**e**) SEM micrographs of bacteria attached to the surface of C_2_G hydrogels and not attached on the surface TC_2_G hydrogels at 24 h.

The result of colony-forming units assay showed that the C_2_G hydrogels demonstrated antimicrobial activity against *P. aeruginosa* and *E. faecalis*, with a reduction of up to 2 log10 CFU/mL compared with the control. The TC_2_G hydrogel in comparison only showed a 1 log10 CFU/mL reduction of *E. faecalis* and no reduction for *P. aeruginosa* (Figure 8a). The live/dead assay showed that TC_2_G hydrogels led to >80% of bacterial death for both strains after 24 h incubation, whilst C_2_G led to 70% death of *E. facaelis* and >80% for *P. aeruginosa* (Figure 8b). Confocal microscopy images of *E. facaelis* and *P. aeruginosa* on the hydrogel surfaces showed that the majority of the bacteria were dead on both hybrid hydrogel types, as indicated by the red stain (Figure 8c). The attachment assay showed the number of bacteria on the C_2_G hydrogel surface was significantly higher than on TC_2_G, with almost all bacteria appearing dead (Figure 8d). This was supported by scanning electron microscopy images showing bacterial adherence on the C_2_G gel, whilst no bacteria appeared adhered to TC_2_G (Figure 8e).

## 3. Discussion

### 3.1. Hybrid Formation

Hydrogels are mainly formed from organic materials and appear to have excellent properties as natural biopolymers for biomedical applications tend to contain the appropriate ligand chemistry. However, the mechanical properties of these hydrogels can be relatively poor and degradation is not predictable. On the other hand, uncontrolled degradation of inorganic hydrogels can hinder stability [51]. Thus, a hybrid hydrogel will give molecularly interpenetrating networks, which are important for maintaining the bioactivity from organic components and mechanical reinforcement from inorganic constituents.

Organic with inorganic crosslinkers such as GPTMS can be employed to overcome uncontrolled degradation. So far, several studies have reported the synthesis of organic-inorganic hybrids using chitosan as the organic source [52,53,54,55,56]. Thiolated chitosan offers advantageous features over unmodified chitosan including significantly improved permeation and mucoadhesive properties arising from thiol groups present on side chains. Thiolated chitosan is also able to form a gel in physiological conditions that facilitates controlled drug release and cell delivery [35,57]. In these hybrid materials, GPTMS was used to cross-link thiolated chitosan and chitosan with inorganic silica derived from a novel silica precursor GLMS. The functionalisation of chitosan (C) and thiolated chitosan (TC) with GPTMS was initially assessed. There are several chemical groups on chitosan for functionalisation with the epoxy ring on GPTMS [49], and functionalisation of two of these groups is more likely to occur using the reaction conditions employed here (Figure 1a). One of the mechanisms proposed utilises covalent coupling of the epoxide ring to the primary amine to form a secondary amine [58,59,60], whilst the other proposed covalent coupling is between the hydroxyl groups of the chitosan via the epoxide ring [61]. Also, during the reaction, the epoxide ring-opening reactions of GPTMS can proceed according to two mechanisms. If the epoxide reacts in basic condition, nucleophiles attack the less substituted carbon while in acidic condition; nucleophiles attack the more substituted carbon to open the epoxide ring and the epoxide ring open to form a diol, (Figure 1a).

A schematic diagram of the silica-organic network in hydrogels is shown in Figure 1b. Chemical characterization (FTIR and ^1^H NMR) confirmed that the hydrogels formed Class II interactions via GPTMS. FTIR spectra of thiolated chitosan prepared in this study did not show any differences compared with unmodified chitosan, this may have been due to the presence of relatively small amounts of thiols. However, the protocol adopted was published previously by Bernkop-Schnürch [62] who showed complete characterisation of the functionalised polymer. Furthermore, functionalised chitosan was soluble in water while unmodified chitosan required an acidic pH to become soluble. Additionally, the NMR spectrum of thiolated chitosan showed a chemical shift for the H-2 proton suggesting the apparent successful functionalisation of chitosan with thiol groups.

Varghese et al. (2010) hypothesised that the epoxide ring would react with the –OH groups of the chitosan and this may be observed for the CG hybrid due to the protonation of amine in chitosan at acidic pH [61,63], however, others have conclusively shown that the reactions of amine with the epoxy group on GPTMS occurred at acidic pH [49]. However, it was likely that a mixture of both reactions occurred in the conditions employed here [64]. The higher pH of the reaction between thiolated chitosan and GPTMS compared with chitosan and GPTMS would have increased the chances of an epoxied reaction with the amine group in thiolated chitosan (Figure 1a). However, it should be noted that there remained a possibility that the reaction may have occurred through hydrogen bonding between amine, amide or hydroxyl species and epoxide groups or ionic bonding between the positively charged amine groups and negatively charged silanes. In addition, the condensation of the silanol groups on the opposing end of the GPTMS molecules was also observed to occur simultaneously. This highlights the dual reactivity of GPTMS and adds further complexity to characterising its reactivity and efficiency in coupling [65,66].

### 3.2. Degradation Behaviour

The weight loss of hydrogels showed that the composition of hybrids with a 2:1 organic: inorganic ratio had the slowest degradation rate. Previous studies have reported the organic: inorganic weight ratios to show the greatest influence on degradation behaviour. Increasing the inorganic content resulted in a highly condensed network formed in the hybrid which resulted in decreased degradation [24,67].

To better understand the contributions of hydrolytic dissolution and enzymatic degradation on the breakdown of hydrogels, TC_2_G and C_2_G samples were incubated in PBS with or without lysozyme for 21 days. It has been reported that in the human body, chitosan is mainly degraded by lysozyme which hydrolyses linkages between glucosamine–glucosamine, glucosamine–N-acetyl-glucosamine and N-acetyl-glucosamine–N-acetyl-glucosamine units [68,69]. The present study utilised a concentration of lysozyme corresponding with levels present in human serum [70] and was used to mimic the in vivo degradation; results were compared with those in lysozyme-free PBS.

In the degradation solutions, burst release of silica and chitosan from hybrid hydrogels occurred in the first few days and then the release rate continued to decrease up to 21 days, while most of the glycerol species were released in the first week, which was responsible for the majority of the weight loss from the hydrogels. This possibly indicated relatively weak electrostatic interactions between glycerol and chitosan, while the chitosan matrix crosslinked with the silica which was degraded enzymatically by chitosan chain cleavage. The rate of degradation was found to be dependent on the amount of GPTMS or, in other words, the degree of crosslinking [71]. Chitosan is degradable at a relatively slow rate, however, in the presence of lysozyme, the degradation was accelerated. According to a previous study, the incorporation of glycerol in the hydrogel systems which contained acrylic acid, N-vinyl-2-pyrrolidone affected the degradation rate, as more than 40% glycerol content in the hydrogel caused destruction of the hydrogen bonds between polymer chains, which decreased the cross-linking density of gels [72]. That phenomenon may have explained the rapid degradation of the hydrogels in the present study over the first few days followed by the subsequent slower degradation.

### 3.3. Rheological Properties of Hybrid Hydrogels

Oscillation rheometry was performed to assess kinetics of gel formation, strength and the viscoelastic nature of the hybrid hydrogels. Gelation of the thiolated chitosan hybrid formulations occurred faster compared with chitosan and this agreed with Stefanov et al. [73] who reported that as the degree of chitosan thiolation increased, the gelation time reduced. This was expected as the thiol groups were able to participate in network formation. Both G′ and G” increased rapidly as gelation proceeded; the build-up rate of G′ was much higher than that of G″. This may have occurred due to the formation of an elastic hydrogel from the crosslinked formation between polyglycerol and chitosan/silica. The transition of the chitosan/silica/glycerol system from a liquid-phase to a solid-phase revealed viscoelastic behaviour and also suggested the formation of a three-dimensional (3D) network [74]. For applications that require hydrogels with reduced gelation times, e.g., for use in the surgical application, TC_2_G potentially provides a useful hybrid hydrogel. Indeed, the advantages of short gelation times avoids excessive drug diffusion in delivery systems or heterogeneous cell distribution within the matrix [75,76,77,78]. Of particular interest was that both gels at different time-points exhibited an identical modulus at the crossover-point indicating a critical strength above which both gels behaved as solids and the network on these materials could be similar or identical to each other.

The effect of the strain amplitude on the hydrogels showed beyond the linear viscoelastic region (LVR) that the elastic modulus abruptly decreased (Figure 4b) indicating that structural breakdown occurred as a consequence of the large deformations imposed. A constant G′ with increasing strain corresponds with an elastic gel which when G′ begins to decrease or exhibit a crossover with G″ and suggested the gel had become more viscous [79]. TC_2_G showed an increase in G′, which represented the elastic component of the materials deformation and was correlated with the number of effective intermolecular cross-links formed in the hydrogel network and consequently, the extension of LVR [77]. At high frequency, G′ was higher than G″. G′ indicated a stronger gel network so TC_2_G had improved the strength of hydrogel than C_2_G. At low frequency, G″ was higher than G′ and this property was related to the relaxation time of the molecular chains. At low frequency, relaxation was greater and the chains could relax more slowly, reducing G′ and G″ [80]. With time the hybrid hydrogels demonstrated increased storage modulus; this was due to the continued polycondensation of the silica network in the hydrogel often seen during the ageing phase of sol-gel materials. A comparison between the chitosan-based hydrogels (2.5 wt%) produced here and gelatin methacryloyl hydrogels (GelMA) (5 wt%) reported in the literature [81] was made. The G′ in a frequency sweep test was reported to be 275 ± 10 Pa [81] whilst the Class II silica-chitosan hybrid hydrogels, in particular, TC_2_G had higher G′ (790 Pa) while C_2_G was relatively similar to GelMA (202 Pa).

Tanδ of TC_2_G was higher than C_2_G, therefore the structures of TC_2_G were considered to be strong. Due to these observations, these hydrogels were considered as stable and strongly crosslinked gels [82]. This stiffening and more solid-like behaviour was caused by the inability of longer polymer chains to rearrange in the given time scale. Thus, TC_2_G had a more “solid-like” structure and offered the potential for tailoring mechanical performance to meet the demands of specific applications.

### 3.4. Hybrid Hydrogels As a Drug Delivery System

To assess the ability of hybrid hydrogel to be used as a drug delivery system, a range of different molecular weight proteins including human OPG (20 kDa), fibronectin (220 kDa), and insulin (5.7 kDa) were selected and loaded into the hydrogels. In general, protein loading efficiencies decreased as protein molecular weight increased. However, high loading efficiencies for different molecular weights in TC_2_G and C_2_G were identified as the total loading protein was released in one week. This implied that molecular weight was not the only factor influencing protein loading and release as no significant difference between different molecular weight proteins released from hydrogels was observed. Protein charge is also identified as a major factor in protein loading efficiency [83]. The amino groups on the chitosan can bind with hydrogen ions to be protonized and consequently exhibit positive charges. At pH 6.5, positively charged chitosan was loaded with negatively charged proteins through electrostatic interactions. Various protein release profiles could be altered by the electrostatic interactions between chitosan and the proteins [84].

Insulin, fibronectin and OPG support a negative net charge at neutral pH values [85,86] enabling loading in chitosan particles more efficiently and providing mucoadhesive properties for adsorption of proteins. The hydrogel developed in this study contained a relatively large amount of water and had less polymeric mass, which was favourable for high-concentration drug loading. However, this can lead to rapid drug release as a result of high mobility of drug molecules in solution within the hydrogel. Our hydrogels were prepared in solutions with a physiological pH, allowing the safe incorporation of bioactive molecules for a broad range of medical applications, particularly for in vivo drug release.

### 3.5. Cell-Material Interactions

The effect of different ratios of organic-inorganic hybrids on the viability of cells has not previously been examined. Our data showed that there was no significant difference between different organic-inorganic ratios on the viability of osteoblasts. The osteoblast viability after encapsulation of cells in the hydrogels was similar at different time-points and the hydrogels did not appear to exert a significant harmful effect on cell viability. Based on previous studies, cytotoxicity responses were classed as slightly cytotoxic when the percentage cell viability was 60–90% [87,88]. Interestingly, cell viability increased with time in direct and indirect assays and was not affected by the hydrogel after 7 days. These observations indicated that the hybrid hydrogel was not toxic and supported cell growth. Cell viability in TCG hydrogels was higher than in C_2_G hydrogels which may be explained by the water-soluble chitosan (thiolated chitosan) not requiring an acidic vehicle for solubilisation whilst chitosan required an acidic environment, which could have been detrimental to cells. Furthermore, incorporation of glycerol into the hydrogel may help to deliver therapeutic agents that enhance cell growth and improve wound healing capabilities of these hydrogels. Indeed, some studies have shown that glycerol incorporation can increase the release of therapeutic agents in the early stages of the release profile by enhancing the formation of water channels [89,90].

In addition, SEM/Cryo-SEM images showed cell attachment and growth on the surface and within the hydrogels. SEM imaging revealed that the hydrogels were soft solids lacking macropores and as previous studies have reported this allowed for dynamic rearrangement of the hydrogel networks to facilitate cell motility [91]. Hybrid hydrogel matrices are water swollen networks of organic and inorganic phases. Therefore, they would exhibit “pores” at the nano to molecular scale. Hydrogel matrices can be either non-porous (having only relatively small pores that are typically in the range of tens of nm for the gel network), or contain macroscopic pores that are typically in the range of 10–500 µm. Dual nano- and macro-porosity is essential for controlled growth of a tissue and drug delivery [92,93,94].

### 3.6. Antibacterial Activities of Hybrid Hydrogels

The antibacterial activities of these hydrogels were tested using *P. aeruginosa* and *E. faecalis* which are the most common bacteria responsible for prosthesis-related infections [95]. The positive charge of chitosan could facilitate electrostatic cross-linking with bacterial membranes, thus enhancing antibacterial activity [96]. Both C_2_G and TC_2_G inhibited growth of *E. faecalis* efficiently; moreover, C_2_G also inhibited *P. aeruginosa*. Additionally, TC_2_G showed an anti-adherent effect which may be explained by a combination of factors, including the reduction of free available amine groups of chitosan, which have previously been related to the adherence of bacteria observed on C_2_G and the increase of surface hydrophilicity, which can decrease bacterial adhesion directly or indirectly through decreased protein adsorption [97]. Therefore, it appears that using thiolated chitosan provides surface characteristics that are interesting for inhibiting bacterial adhesion. Nevertheless, it is not clear if the exposed thiol group had any direct contribution in preventing specific adhesion (e.g., degrading relevant disulfide bridges of bacterial adhesins), or if the overall mechanism was purely non-specific anti-adhesive [97].

### 3.7. Limitations of Study and Future Works

In this study, the effect of hydrogels on viability and mineralisation of osteoblasts in long-term cell culture was not evaluated. This requires further investigation in future studies using cells to study the effect of hydrogels on the differentiation, proliferation and mineralisation capability of cells.

## 4. Materials and Methods

### 4.1. Materials

Low molecular weight chitosan (75–85% deacetylated), 2-iminothiolane hydrochloride and an organosilane (3-glycidyloxypropyl) trimethoxy silane (GPTMS) were purchased from Merck (Darmstadt, Germany) and used in polymer functionalisation. Human recombinant osteoprotegerin (OPG, PEPROTECH, 1 mg, East Windsor, NJ, USA), human recombinant insulin (Sigma-Aldrich, Gillingham, UK), human fibronectin (Sigma-Aldrich, Gillingham, UK) were used for protein release assay. ELISA kits for human insulin, fibronectin and OPG were purchased from R&D Systems (Minneapolis, MN, USA). The human osteosarcoma cell line, SaOs-2 (ATCC HTB-85, Manassas, VA, USA), cell culture medium McCoys (Sigma-Aldrich, Gillingham, UK) supplemented with 10% (*v*/*v*) foetal calf serum (Biosera, Nuaillé, France), 0.297 g L-glutamine/500 mL bottle of media (Sigma-Aldrich, Gillingham, UK), 1% penicillin–streptomycin (PS; 100 IUmL^−1^ penicillin, 0.1 mg mL^−1^ streptomycin (Sigma-Aldrich, UK) were used for cell culture. Resazurin sodium salt (0.3 mg/mL) (Sigma-Aldrich, Gillingham, UK) was used for the Alamar Blue assay and the live/dead two-colour fluorescence assay was purchased from ThermoFisher (Waltham, MA, USA).

### 4.2. Synthesis of Hydrogels

#### 4.2.1. Functionalisation of Chitosan

Thiolated chitosan (TC) was synthesised as previously described [62]. In brief, 5 g of low molecular weight chitosan (C) was dissolved in 500 mL of 1% acetic acid, the pH was adjusted to 6 with 5 M NaOH and reacted with 0.1 mg/mL of 2-iminothiolane HCl (50 mg) for 24 h with continuous stirring at room temperature. The solution was then dialysed against dilute HCl (twice against 5 mM HCl containing 1% NaCl followed by 5 mM HCl and finally against 0.4 mM HCl). Dialysed TC was freeze-dried at −60 °C and 0.4 mbar (Edwards K4 Modulyo Freeze Dryer) and stored at 4 °C until use. Thiolated chitosan was further functionalised with GPTMS before hydrogels were synthesised. Thiolated chitosan (17 mg/mL) was initially dissolved in deionized water and GPTMS was pipetted in at chitosan monomer to GPTMS molar ratio of 4:1 and stirred for 24 h at room temperature [22,49]. Low molecular weight chitosan was functionalised with GPTMS using the same approach, however, the chitosan was dissolved in 1% acetic acid and after reaction with GPTMS, the pH was adjusted to 6.5 with 0.5 M NaOH.

#### 4.2.2. Synthesis of Glycerol-Modified Silane (GLMS) Precursors

Tetraethyl orthosilicate (TEOS; Merck, Germany) was transesterified with dry glycerol (Merck, Germany) with 1:4 (TEOS: glycerol) ratio at 140 °C in an argon atmosphere as previous described [98,99,100]. The reaction was performed until distillation of ethanol stopped. GLMS was stored in a dry atmosphere at room temperature to prevent reaction with moisture.

#### 4.2.3. Hydrogel Formation

GLMS was dissolved in deionized water or cell culture media and added to functionalised chitosan solution in different organic-inorganic weight ratios (1:1, 2:1 and 10:1) to prepare thiolated chitosan-ilica hybrid or chitosan-silica hybrid solutions. The hybrid solutions were allowed to gel under room temperature to form the hydrogels (Thiolated-silica hydrogels: TC_1_G, TC_2_G and TC_10_G or chitosan-silica hydrogels: C_1_G, C_2_G, C_10_G) with 3, 2.5 and 2 wt% respectively. The amount of chitosan/thiolated chitosan, GPTMS and GLMS used to prepare 1 mL of hybrid hydrogels are given in Table 1.

### 4.3. Physio-Chemical Characterisation of Hydrogels

#### 4.3.1. Chemical Characterization of Functionalised Polymer and Hydrogels by FTIR and 1H NMR

The chemical structure of starting materials, functionalised polymers and hydrogels were determined using FTIR in reflection mode (ThermoFisher (Waltham, MA, USA), Nicolet iN10 MX, USA) and 1H NMR (Bruker, AVIII300, USA). Hydrogels were air dried and the FTIR spectra recorded from 400–4000 cm^−1^, at a resolution of 8 cm^−1^ and three scans were performed [101]. 1H NMR measurements were performed in D_2_O solution by dissolving 11 mg chitosan in 650 µL D_2_O and 6.5 µL acetic acid and thiolated chitosan in 650 µL D_2_O. GPTMS was added dropwise to the solutions to produce a chitosan monomer to 6 mg/mL GPTMS ratio of 4:1 and reacted for 24 h at room temperature before performing 1H NMR measurements. Unreacted starting materials including acetic acid, GPTMS, chitosan in acetic acid and thiolated chitosan dissolved in D_2_O were also analysed. Mnova V.14 software was used to analyse NMR spectra.

#### 4.3.2. Rheological Behaviour of the Hydrogels

Rheological studies of the hydrogels were performed using a parallel-plate geometry (40-mm diameter, 1–1.4 mm gap) oscillation rheometer (TA Instruments AR G2, Waters, Milford, MA, USA). The temperature of the plates was fixed at 37 °C using a Peltier-cooled stage. For time-dependent behaviour of hydrogels, the temperature of the Peltier-plate was maintained at 25 °C (room temperature) to mimic gelling conditions of hydrogels prepared in this study. Strain sweep measurements from 0.1 to 1000% at a constant frequency 10 rad/s were performed to determine the strain amplitude in the linear viscoelastic region (LVE). Frequency sweep studies in the LVE region (0.1 to 100 rad/s at a constant deformation of 1% strain) were performed to determine the storage (G′) and loss modulus (G″). An oscillation time sweep was performed to measure and record the evolution of storage and loss modulus (G′ and G″, respectively) over a fixed time period, at an angular frequency of 10 rad/s and at a constant strain of 1%. TRIOS software (TA, version 4.5.1) was employed to analyse the results of rheological tests.

#### 4.3.3. In Vitro Degradation of the Hydrogel

The degradation behaviour of the hydrogels was evaluated by measuring wet weight of the hydrogels (weight of the water-absorbed gel after the excessive fluid is removed from the gel) over time in phosphate buffered saline (PBS, pH 7.4). Hydrogels (10 mg) were placed in polypropylene conical tubes (Cole-Parmer Instrument Company LTD, Eaton Socon, UK), each containing either 5 mL PBS or 5 mL PBS containing 1.5 mg/mL lysozyme. The PBS solutions were replaced with fresh solutions daily. This ensured active lysozyme was present in the media [70,87]. The hydrogel sample and solution were sealed and maintained at 37 °C with mild agitation (50 rpm) for the duration of the study. Chitosan-free inorganic hydrogels (GLMS hydrogel) (10 mg) prepared by dissolving 0.1 g GLMS in 1mL water at 10 wt% were used for comparison. Inductively coupled plasma atomic emission spectroscopy (ICP-AES, PerkinElmer, Waltham, MA, USA) was used to obtain the soluble silica release profiles in PBS solution and high-performance liquid chromatography (HPLC, Shimadzu, Wolverton, UK) was used for the quantification of glycerol and glucose in degradation solutions. Experiments were performed in triplicates [102,103].

### 4.4. Protein Release from Hybrid Hydrogels

Hydrogels containing OPG, insulin and fibronectin were also prepared by dissolving 17 µg/mL of protein with GLMS in deionized water to prepare the hydrogel as described previously. OPG concentration was selected based on previous in vitro and in vivo studies [87,104,105]. Insulin and fibronectin concentrations were also kept at 17 µg/mL to ensure consistency. Hybrid hydrogels (10 mg) containing insulin, fibronectin and OPG were placed in tubes with 5 mL of PBS (pH 7.4). Samples were agitated at 40–50 horizontal strokes per minute at 37 °C, the supernatant was collected and replaced with fresh PBS solution at a range of time intervals from 1–7 days. The samples were centrifuged and filtered and the protein release from the gel was analysed by using ELISA for human insulin, fibronectin and OPG following manufacturer’s instructions. Experiments were performed in triplicate and the means calculated. The amount of protein added into 10 mg of hydrogel was 40 mg, consequently the percentage of protein released from the hydrogel was calculated by dividing the amount of protein released in a specific time by the total protein.

### 4.5. Assessment of Cell-Material Interactions

#### 4.5.1. Culture of Osteoblasts (SaOs-2) with Hydrogels

The cytotoxicity of hydrogels was evaluated by both direct and indirect contact methods using SaOs-2 cells which is a cell line derived from the primary human osteosarcoma. The reagents used to prepare the hydrogels were sterilized using 254 nm UV light (UV Steriliser Cabinet: Adexa, Hamburg, Germany) for 1 h. The solutions were cast into culture 24 wellplates or petri dishes (Ibidi, Gräfelfing, Germany) and gelled under aseptic conditions in a class II biological safety cabinet (Monmouth Scientific, Bridgwater, UK). Cells were cultured at 37 °C in a humidified atmosphere of 5% CO_2_ in air, in McCoys media. The medium was supplemented with 10% foetal bovine serum (FBS) with 1% antibiotics (penicillin-streptomycin) and incubated in 5% CO_2_ at 37 °C. All assays were performed in triplicate.

#### 4.5.2. Indirect Contact Method

The hydrogels were incubated with McCoys media for 24 h in 0.2 g/mL hydrogel sample to media ratio according to international standard number ISO 10993-12 [106]. Cells were cultured at a density of 2 × 10^4^ cells per well in a 48-well culture plate for 24, 48 and 72 h in media extracted from hydrogels. Cells cultured in extract-free McCoys media were used as controls. Subsequently, the medium was gently aspirated from the culture wells and replaced with 10% AlamarBlue reagent to determine cell viability. In the Alamar Blue Reagent assay, the growing cells resulted in a chemical reduction of the Alamar Blue dye from blue to red which can be detected using a fluorescence absorbance detector. Cultures were incubated with the AlamarBlue reagent for 5 h in culture conditions and 100 µL media were collected in a 96-well plate for detection of absorbance at excitation of 570 nm and emission 600 nm using a microplate reader (Spark 20M Multimode, Tecan, Männedorf, Switzerland) [107].

#### 4.5.3. Direct Contact Method

Hydrogels were prepared following method described in Section 4.5.1 and osteoblasts (SaOs-2) were seeded at a density of 2 × 10^4^ cells per well on the surface of the hydrogels in a 48-well culture plate. Cells seeded on the hydrogel-free 48-well plate were used as control. After 24, 48, 72 and 168 h, cell viability was determined using the AlamarBlue assay following the method described in Section 4.5.2.

#### 4.5.4. Live/Dead Assessment of Cell Encapsulated or Seeded on Hydrogels

The hybrid hydrogel was prepared as previously described in Section 4.2.3 by mixing GLMS dissolved in deionized water and then adding the functionalised chitosan solution. SaOs-2 cells were then added to the solution by mixing 100 µL of the SaOs-2 suspension with 900 µL of solution to achieve a cell density of 1-million cells/mL. From this 200 μL of the mixture was pipetted into 35 mm petri dishes to cover a 21 mm area with 1 mm height. A fluorescence assay (LIVE/DEAD) was employed to determine the cell viability in hydrogels at 24, 48, 72 and 168 h of culture. The LIVE/DEAD assay kit contains two fluorescence dyes, calcein-AM, which stains the live cells, and ethidium homodimer-1 which stains the dead cells. Hydrogels were washed in PBS prior to staining. 100–150 μL of the combined LIVE/DEAD assay reagent was added to the surface of the hydrogel. The hydrogels containing the staining agent were allowed to rest for 45 min in the dark. A Zeiss LSM 700 confocal microscope (Zeiss, Jena, Germany) was used for image acquisition and ImageJ software was used for analysis [108]. Cell viability was calculated as the number of Live cells (green)/number of total cells and expressed as a percentage.

#### 4.5.5. Scanning Electron Microscopy (SEM) of Hydrogels Containing Cells

The hydrogels containing cells were imaged at 168 h after encapsulation using conventional SEM and Cryo-SEM. For SEM, cell encapsulated hydrogels were washed in PBS and fixed in 2.5% glutaraldehyde in cacodylate buffer for 30 min. Gels were dehydrated through a graded series of ethanol from 20%, 70%, 90%, twice in 95%, and twice in 100% for 10 min each. Finally, samples were placed in 50:50 in ethanol and 100% hexamethyldisilizane and allowed to evaporate overnight in a fume cupboard. Once dry, samples were gold sputter coated at 20 mA for 3 min, to produce a ~20 nm thick coating using an EM-Tec sputter coater (Emitech K550X, Quorum Technologies, Lewes, UK). Samples were then imaged with a Zeiss EVO/MA10 scanning electron microscope (Zeiss, USA) using an accelerating voltage of 10 kV [109]. Wet hydrogels were imaged using Cryo-SEM. Hydrogels were frozen by plunging into liquid nitrogen and freeze-fractured in the cryo-preparation chamber and cryo-SEM micrographs were obtained using an XL30 FEG ESEM with an accelerating voltage of 5 kV [110].

### 4.6. Antibacterial Effects of Hydrogels

The hydrogels were assessed for their in vitro antibacterial activity against two clinical species of bacteria, including the Gram-positive *Enterococcus faecalis* (*E. faecalis*) (ATCC 29212) and the Gram-negative *Pseudomonas aeruginosa* (*PA14*). Colony-forming units, adherence assay, and live/dead assay were used to evaluate the antimicrobial properties (Figure S2).

#### 4.6.1. Colony-Forming Units

Two representative, clinically relevant bacterial species were used in this study. A single colony grown on Tryptic Soy Agar was inoculated in 10 mL of Tryptic soy broth (TSB) (Oxoid, Basingstoke, UK) and left to grow over-night at 37 °C in a shaking incubator (NB-205, N-Biotek, Wonmi-gu, Korea). After 24 h, 100 µL of bacteria was diluted in 900 µL TSB and the optical density (OD) was read using a 7315 Spectrometer (Jenway, Stone, UK). The over-night cultures were diluted to an optical density of (0.1) and TSB without bacteria was used as standard. 1 mL of the diluted cultures was added on top of a hydrogel prepared in a 24-well plate and then incubated at 37 °C for 24 h. After the exposure of the bacteria to the samples, the quantification of viable bacteria in broth was performed using the Miles and Misra Method. Plates were incubated at 37 °C for 24 h (Heracell 150i, ThermoFisher, Waltham, MA, USA), and then the numbers of colonies were counted. These results were compared with the number of colony forming units of the untreated control group, which had not been exposed to the hydrogel samples.

#### 4.6.2. Adherence Assay

The hydride hydrogels were prepared on 24 well plates. 1mL of the overnight bacterial suspension was cultured on the hydrogels (0.1 OD) at 37 °C in a shaking incubator. After 24 h, hydrogels with attached bacteria were removed from the well and washed with PBS to remove loosely adhered bacteria. To observe the bacteria on the hydrogel, live/dead staining and SEM imaging were performed.

#### 4.6.3. Live/Dead Viability Assay

A LIVE/DEAD assay was employed to determine the live/dead percentage of bacteria seeded on hydrogels after 24 h. 3 μL of SYTO^®^ 9 stain and 3 μL of propidium iodide stain were mixed in 1 mL of sterilized PBS. 200 μL of staining solution was added to the surface of the hydrogel. The hydrogels containing the staining agent were stained for 30 min in the dark. Zeiss LSM 700 confocal microscope was used for image acquisition and ImageJ software was used for analysis. The percentage of dead bacteria was calculated (number of red stained cells/number of total cells) × 100%.

### 4.7. Statistical Analysis

Statistical analysis was performed using parametric One-Way ANOVAs for comparison between the mean values of different groups. The viability percentage of osteoblasts were analysed using independent-sample *t*-tests.

## 5. Conclusions

The hydrogels demonstrated specific degradation and mechanical properties that supported the growth of cells, which may be used for specific applications such as tissue engineering. The hybrid hydrogels exhibited high protein loading efficiencies and delivered different molecular weight proteins over a week, which indicated a potential use as delivery systems for a range of different molecular weight proteins/molecules. The hydrogels showed no significant cytotoxic effects during material-cell contact indicating the hydrogel could be used to support the growth of cells. Our hydrogels demonstrated antimicrobial activity against *P. aeruginosa* and *E. faecalis* leading to a potential reduction in medical application related infections. It is suggested that these newly developed TC_2_G and C_2_G hydrogels have both inorganic and organic components that could provide inherent bioactivity due to the exposure of both inorganic and organic components to host tissue/cells. In conclusion, it appears that the TCG2/CG2 hybrid hydrogels show potential for the delivery of cells and therefore for possible use in bone tissue engineering, drug and cell delivery systems.

## Figures and Tables

**Table 1:** The amounts of chitosan/thiolated chitosan, GPTMS and GLMS used in a 1 mL hydrogel.

Hydrogel	Chitosan/Thiolated Chitosan (mg)	GPTMS (mg)	GLMS (mg)
TC_1_G/C_1_G	17	6.25	100
TC_2_G/C_2_G	17	6.25	50
TC_10_G/C_10_G	17	6.25	10

## Data Availability

Data is contained within the article or Supplementary Materials.

## Supplementary Materials

The following are available online at www.mdpi.com/1422-0067/222/21/2267/s1.
