# Supplementary material for: Novel Chitosan-Silica Hybrid Hydrogels for Cell Encapsulation and Drug Delivery
Source: Int J Mol Sci. 2021 Nov 12;22(22):12267. doi: 10.3390/ijms222212267 (PMC8624171; doi:10.3390/ijms222212267)
Supplement: Supplementary file 1 [file ijms-22-12267-s001.zip › ijms-1436377-supplementary.pdf]

Supplementary Materials: The following are available online at www.mdpi.com/1422-0067/222/21/2267/s1.

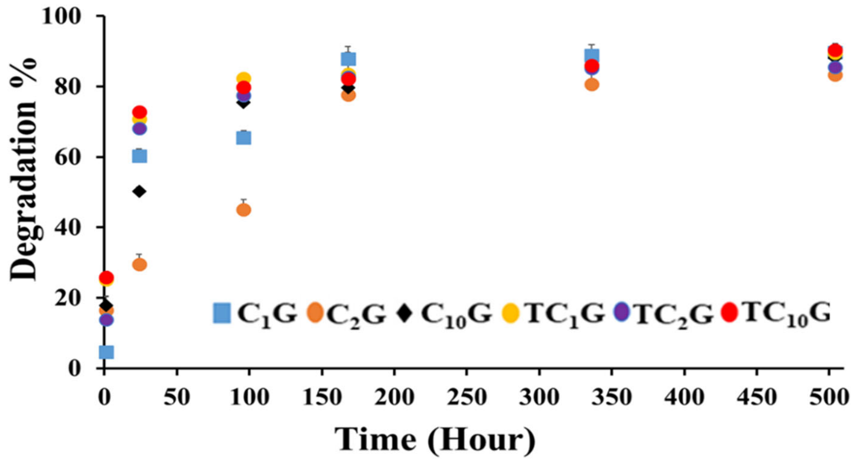

Figure S1: Degradation percentages for the different types of hydrogels after 504 hours showing the slowest degradation rate in C_2G and TC_2G.

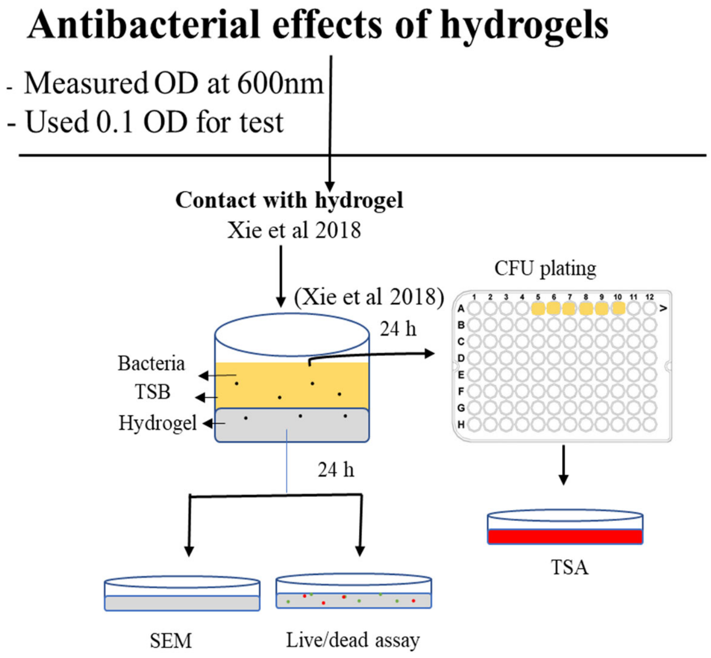

Figure S2: Schematic showing the assays used to evaluate the antibacterial activity of hydrogels. CFU: colony-forming unite, TSB: Tryptic soy broth, TSA: tryptone soya agar, SEM: scanning electron microscope.

Table S1. Silica release from the different types of hydrogels at 0.5, 1, 7, 24, 96, 168, 336, 504 hours. Significant differences were identified through one-way ANOVAs and post-hoc Tukey comparisons ($p<0.05$). Lower case letters represent the post-hoc Tukey comparisons at each time (across the rows) where the significantly highest to lowest release is represented in ascending alphabetical order.

Hydrogels / Time (hour)	C2G BPS	C_2G E	TC_2G PBS	TC_2G E
**0.5**	$0.88±0.1^{bc}$	$3.04±0.39^{a}$	$0.77±0.17^{c}$	$0.97±0.18^{b}$
**1**	$0.869±0.09^{b}$	$1.29±0.54^{a}$	$0.55±0.13^{c}$	$0.76±0.08^{b}$
**7**	$5.0±0.26^{b}$	$7.84±0.2^{a}$	$2.83±0.35^{d}$	$1.94±0.24^{c}$
**24**	$3.05±0.37^{a}$	$1.11±0.17^{c}$	$2.46±0.62^{b}$	$2.69±0.26^{b}$
**96**	$0.79±0.05^{d}$	$1.55±0.08^{c}$	$2.04±0.08^{a}$	$1.87±0.06^{b}$
**168**	$1.11±0.9^{d}$	$1.31±0.21^{b}$	$1.28±0.013^{c}$	$1.41±0.1^{a}$
**336**	$1.42±0.1^{d}$	$1.96±0.1^{b}$	$1.67±0.01^{c}$	$2.08±0.12^{a}$
**504**	$0.95±0.10^{d}$	$1.58±0.25^{b}$	$1.18±0.06^{c}$	$1.70±0.14^{a}$

Table S2: Glycerol release from the different types of hydrogels at 0.5, 1, 7, 24, 96, 168, 336, 360, 504 hours. Significant differences were identified through one-way ANOVAs and post-hoc Tukey comparisons ($p<0.05$). Lower case letters represent the post-hoc Tukey comparisons at each time (across the rows) where the significantly highest to lowest release is represented in ascending alphabetical order.

Hydrogels / Time (hour)	C2G BPS	C2G E	TC2G PBS	TC2G E
**0.5**	217.45± 37[b]	248.53±30[a]	169.78±31[c]	172.53±32[c]
**1**	166.4±28[b]	169.35±39[a]	162.15±29[c]	162.65±25[c]
**7**	136.25±31[a]	136.83±10[a]	100.15±16[c]	121.5±21[b]
**24**	80.33±19[a]	80.7±17[a]	79.33±9[a]	79.55±11[a]
**96**	227.03±40[a]	228.68±44[a]	221.4±45[d]	225.05±45[b]
**168**	197.48±20[b]	198.53±31[a]	193.08±23[d]	195.75±41
**336**	140.4±35[b]	146.9±29[a]	124.95±36[d]	134.73±23[c]
**360**	4.38±1[b]	4.975±0.5[a]	2.875±0.4[d]	3.975±0.8[c]
**504**	0	0	0	0

Table S3: Chitosan monomers release from the different types of hydrogels at 0.5, 1, 7, 24, 96, 168, 504 hours. Significant differences were identified through one-way ANOVAs and post-hoc Tukey comparisons ($p<0.05$). Lower case letters represent the post-hoc Tukey comparisons at each time (across the rows) where the significantly highest to lowest release is represented in ascending alphabetical order.

Hydrogels / Time (HOUR)	C2G BPS	C2G E	TC2G PBS	TC2G E
**0.5**	2.62±0.43^{b}	3±0.2^{a}	1.83±0.1^{d}	1.97±0.37^{c}
**1**	1.20±0.1^{b}	1.31±0.13^{a}	0.78±0.14^{d}	1.04±0.12^{c}
**7**	0.66±0.13^{ab}	0.69±0.13^{a}	0.62±0.06^{c}	0.63±0.03^{bc}
**24**	0.57±0.14^{a}	0.58±0.13^{a}	0.46±0.41^{b}	0.49±0.01^{b}
**96**	1.14±0.12^{ab}	1.17±0.24^{a}	1.03±0.7^{b}	1.12±0.17^{ab}
**168**	0.64±0.13^{ab}	0.65±0.13^{a}	0.6±0.09^{b}	0.61±0.01^{bc}
**336**	1.09±0.13^{a}	1.19±0.12^{a}	1.06±0.11^{a}	1.12±0.15^{a}
**504**	0.81±0.1^{ab}	0.86±0.11^{a}	0.77±0.07^{b}	0.79±0.03^{b}

Table S4: Effect of the different hydrogels on metabolic activity of osteoblasts at 24, 48, 72 and 168 hours. Significant differences were identified through one-way ANOVAs and post-hoc Tukey comparisons ($p<0.05$). Lower case letters represent the post-hoc Tukey comparisons at each time (across the rows) where the significantly highest to lowest release is represented in ascending alphabetical order.

Hydrogels / Time (HOUR)	C_1G	C_2G	$C_{10}G$	TC_1G	TC_2G	$TC_{10}G$
**24**	49.01±2.9^{a}	48.09±1.8^{a}	49.15±9.5^{a}	51.33±5.9^{a}	51.89±4.06^{a}	51.58±3.5^{a}
**48**	42.10±7.0^{b}	59.58±6.1^{ab}	59.68±10.4^{ab}	66.41±12.3^{ab}	63.48±10.3^{ab}	68.88±5.6^{a}
**72**	57.35±3.1^{ab}	68.01±1.8^{a}	52.41±11.4^{b}	69.54±7.5^{a}	64.09±6.4^{ab}	71.49±3.7^{a}
**168**	61.69±3.3^{b}	71.93±8.3^{ab}	65.37±10.8^{ab}	79.43±8.9^{a}	79.59±2.8^{ab}	77.18±8^{ab}
